# Arp2/3 complex and the pentose phosphate pathway regulate late phases of neutrophil swarming

**DOI:** 10.1016/j.isci.2023.108656

**Published:** 2023-12-16

**Authors:** Katharina M. Glaser, Jacob Doon-Ralls, Nicole Walters, Xilal Y. Rima, Angelika S. Rambold, Eduardo Réategui, Tim Lämmermann

**Affiliations:** 1 Max Planck Institute of Immunobiology and Epigenetics, 79108 Freiburg, Germany; 2 International Max Planck Research School for Immunobiology, Epigenetics and Metabolism (IMPRS-IEM), 79108 Freiburg, Germany; 3 Faculty of Biology, University of Freiburg, 79104 Freiburg, Germany; 4 William G. Lowrie Department of Chemical and Biomolecular Engineering, The Ohio State University, Columbus, OH, USA; 5 Comprehensive Cancer Center, The Ohio State University, Columbus, OH, USA; 6 Institute of Medical Biochemistry, Center for Molecular Biology of Inflammation (ZMBE), University of Münster, 48149 Münster, Germany

**Keywords:** Immunology

## Abstract

Neutrophil swarming is an essential process of the neutrophil response to many pathological conditions. Resultant neutrophil accumulations are hallmarks of acute tissue inflammation and infection, but little is known about their dynamic regulation. Technical limitations to spatiotemporally resolve individual cells in dense neutrophil clusters and manipulate these clusters *in situ* have hampered recent progress. We here adapted an *in vitro* swarming-on-a-chip platform for the use with confocal laser-scanning microscopy to unravel the complexity of single-cell responses during neutrophil crowding. Confocal sectioning allowed the live visualization of subcellular components, including mitochondria, cell membranes, cortical actin, and phagocytic cups, inside neutrophil clusters. Based on this experimental setup, we identify that chemical inhibition of the Arp2/3 complex causes cell death in crowding neutrophils. By visualizing spatiotemporal patterns of reactive oxygen species (ROS) production in developing neutrophil swarms, we further demonstrate a regulatory role of the metabolic pentose phosphate pathway for ROS production and neutrophil cluster growth.

## Highlights

• Swarming-on-a-chip platform was adapted for confocal laser-scanning microscopy
• Live visualization of subcellular components in single cells of neutrophil clusters
• Inhibition of the Arp2/3 complex causes blebbing and death of crowding neutrophils
• ROS production and neutrophil cluster growth are metabolically regulated

## Graphical abstract

**Figure:**
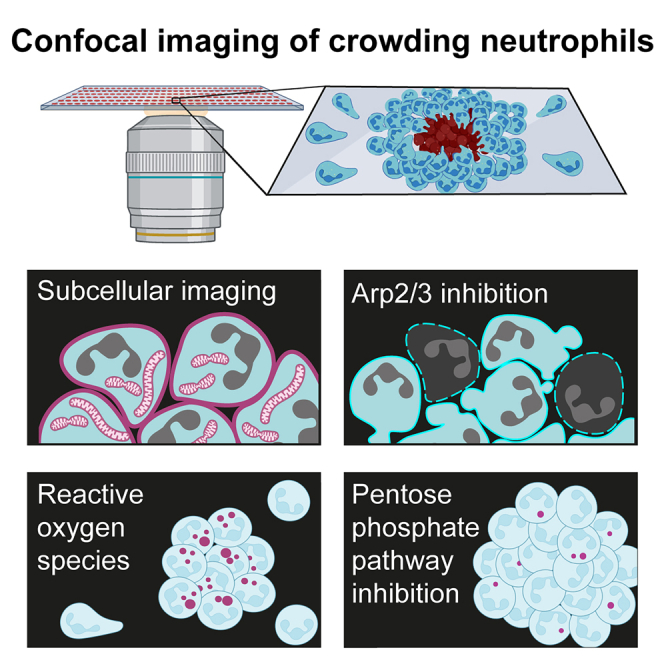

## Introduction

Neutrophils, key immune cells for eliminating bacteria and fungi, accumulate as one of the first blood-recruited inflammatory cells at local tissue injury and infection sites. More than a decade ago, intravital imaging studies revealed a surprising migratory behavior of neutrophils in wounded or microbe-infected mouse tissues^1^: Many individual neutrophils switched from random motility to coordinated chemotaxis, triggering the focal accumulation of these cells and the formation of neutrophil clusters, sometimes also referred to as aggregates. Since then, this series of sequential phases, termed neutrophil swarming, has been observed in more than 30 inflammatory conditions, ranging from sterile inflammation to infections with bacteria, parasites, viruses, and fungi.^2^^,^^3^^,^^4^ Intercellular communication among neutrophils is central to the swarming response and mediated by the release of the lipid leukotriene B4 (LTB4). External stimulants, which substantially elevate intracellular calcium levels, can trigger LTB4 secretion by neutrophils within a few minutes.^5^ When few individual neutrophils detect sites of tissue or cell damage, they can rapidly release LTB4 to attract a second wave of neutrophils. This initiates a positive amplification mechanism to recruit more neutrophils from distant sites before cells crowd and form stable multicellular clusters to isolate the local site of injury or infection from healthy tissue.^5^ Studies on swarming neutrophils from mice,^6^ zebrafish,^7^^,^^8^ and humans^9^ confirmed the crucial role of LTB4-mediated auto-signaling in this process across different vertebrate species.

Different imaging-based experimental systems have identified mechanistic aspects of the dynamic neutrophil swarming response. Intravital imaging of gene-manipulated and transgenic neutrophils in mouse and zebrafish tissues allowed important insight into molecular pathways that control the early phases of neutrophil swarming.^6^^,^^7^^,^^8^^,^^10^ In particular, these studies were crucial in dissecting the self-organizing nature of neutrophil swarms in complex cellular environments. The auto-amplifying activity of neutrophils could be identified for shaping early phases of swarming.^6^^,^^7^^,^^8^ Recently, imaging of neutrophils in mouse tissues discovered a cell-intrinsic stop mechanism contributing to the termination of neutrophil swarming.^11^ As intravital microscopy techniques illuminate fluorescent cells in light-scattering tissue of varying depths, they have limited optical resolution. When cells are dispersed in the tissue during the early phases of swarm initiation and amplification, single neutrophils can still be discriminated for cell tracking analysis. However, this is often impossible once cells enter the fluorescent mass of crowding neutrophils during the late phases of swarming.

Systematic studies on neutrophil swarming outside the living animal have long been problematic. Although specific experimental conditions were known to induce LTB4-dependent neutrophil clustering in a culture dish,^12^^,^^13^ such setups did not allow the time-resolved analysis of sequential swarming phases. Technical developments in biochemical engineering led to the establishment of microfabricated devices for replicating neutrophil swarming dynamics *in vitro*. In these assay systems, neutrophils are exposed to micropatterned arrays of external stimulants, such as zymosan or heat-killed bacterial particles, which can trigger LTB4 secretion and swarming of neutrophils.^5^^,^^9^^,^^14^ These swarming-on-a-chip platforms have initially been established for studies on the swarming behavior of human neutrophils,^9^^,^^15^ whose migration dynamics cannot be studied in living tissues. Several studies have used these controllable swarming devices for the functional characterization of human neutrophils from the blood of patients with different disease backgrounds.^9^^,^^16^^,^^17^^,^^18^^,^^19^ Furthermore, technical adaptations now also allow the study of neutrophil swarming in response to patterned arrays of living microbes, in particular different species of living fungi.^18^^,^^20^^,^^21^^,^^22^ Thus, recent developments of swarming-on-a-chip platforms have brought forward versatile applications for the study of primary human and mouse neutrophils, but also neutrophil-like cell lines.^23^ Several biological findings were derived from such studies, including the analysis of the neutrophil swarm secretome,^9^ the involvement of exosomes in swarming,^24^ the interaction of swarming neutrophils with monocytes,^25^ and the functional mapping of molecular pathways involved in the neutrophil swarming response *in vitro.*^11^^,^^16^^,^^18^^,^^21^^,^^26^ Although several of these studies performed live-cell imaging to capture neutrophil swarming dynamics, the use of bright-field and widefield fluorescence microscopy at low resolution prevented the visualization of more detailed biological information, e.g., subcellular components inside individual cells. By using fluorescent calcium sensors, Khazen et al. and Strickland et al. could recently demonstrate that transient waves of calcium signals travel from individual neutrophils in the center to neighboring cells during the development of a neutrophil cluster. These elegant studies showed the power and great potential of applying fluorescence microscopy techniques to studies on *in vitro* swarming.^16^^,^^27^ Despite all these technical developments, the analysis of crowding neutrophils during the late stages of swarming has been similarly problematic in swarming-on-a-chip platforms as in native tissues. Experimental *in vitro* setups were also limited in spatiotemporally resolving individual cells in dense neutrophil clusters. Due to these constraints, we still have a very limited understanding of the mechanisms underlying the dynamic processes of neutrophil cluster formation.

To overcome current experimental limitations, we here present a technically adapted swarming-on-a-chip platform, which can be used in combination with live-cell confocal laser-scanning microscopy (CLSM). Confocal sectioning of dynamic neutrophil clusters allowed the identification of single cells and the visualization of intracellular components at subcellular resolution. We present examples of how this methodology can help studying the dynamics of mitochondria, cell membranes, cortical actin, phagocytic cups, and reactive oxygen species (ROS) activation in populations of crowding neutrophils. Such information should prove useful in dissecting the functional contribution of single-cell responses to the population response of a whole neutrophil swarm. By using this improved experimental setup, we show a functional role of the Arp2/3 complex for maintaining cell integrity in crowding neutrophils. Furthermore, we demonstrate that the metabolic pentose phosphate pathway (PPP) controls ROS production in crowding neutrophils, which has consequences for neutrophil cluster growth. Our results show that this refined *in vitro* system allows the rapid testing and identification of functional subcellular processes during neutrophil swarming, which are worth further exploration in living organisms.

## Results

### Visualizing the dynamics of crowding neutrophils in the dish

**Figure 1:**
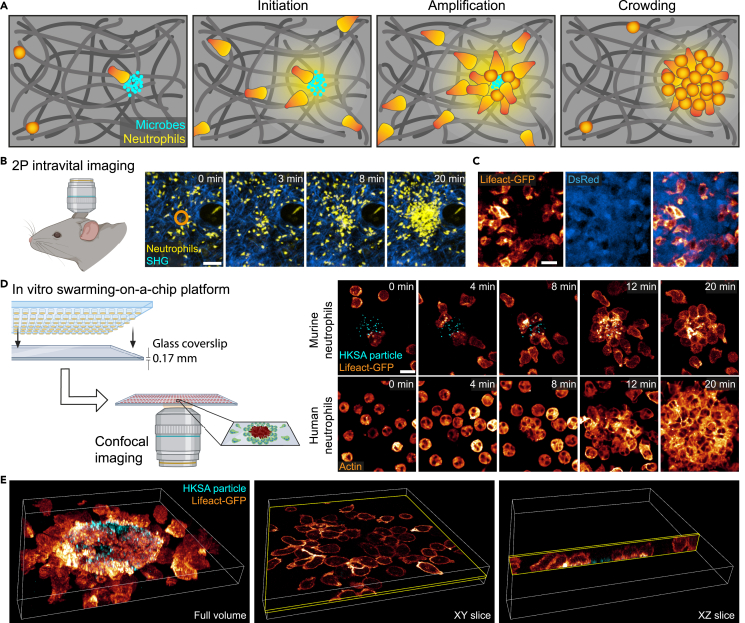
Visualizing the dynamics of crowding neutrophils *in vivo* and *in vitro* (A) Scheme of the sequential phases of neutrophil swarming in tissues. (Outer left) Pioneer neutrophils are attracted to a tissue lesion. (Inner left) They release swarm chemoattractants to recruit more neutrophils, leading to an (inner right) amplified recruitment of neutrophils from the surrounding. (Outer right) Many neutrophils undergo crowding at the lesion site and form neutrophil aggregates. (B) Two-photon intravital imaging (2P-IVM) of swarming neutrophils *in vivo*. Dye-labeled mouse neutrophils (yellow) were injected into the mouse ear dermis. A focal wound was induced 3 h later by a laser beam (orange circle), and neutrophil swarming recorded in an anesthetized mouse. Second harmonic generation (SHG) signal (blue) visualizes fibrillar collagen in the dermis. Scale bar: 50 μm. (C) 2P-IVM of crowding neutrophils in the late phase of neutrophil swarming in the mouse ear skin. Injected Lifeact-GFP-expressing neutrophils (glow) formed cell aggregates with endogenous neutrophils expressing cytosolic dsRed (blue). Scale bar: 25 μm. (D) Swarming-on-a-chip platform with defined patterns of heat-killed *S. aureus* particles (cyan) on thin glass cover slips allows confocal laser-scanning microscopy (CLSM) of swarming neutrophils *in vitro.* Right upper images: Time course of swarming Lifeact-GFP-expressing mouse neutrophils (glow heatmap displaying fluorescent intensity; merged projection of z stack; upper row). Right lower images: Actin stainings of swarming human neutrophils over time (glow). Scale bars: 10 μm. (E) CLSM allows 3D visualization of swarming and crowding mouse neutrophils. z stack of 9 μm with 16 focal planes (left). Single slices in *x-y* (middle) and *x-z* dimension (right). See also Figure S1 and Video S1. Swarming of mouse neutrophils in a swarming-on-a-chip platform, related to Figure 1D, upper row, Video S2. Neutrophil crowding on differently sized HKSA patterns, related to Figure S1C, Video S3. Confocal 3D acquisition of crowding neutrophils, related to Figure 1E.

As introduced, neutrophil populations in inflamed and infected tissues can form multicellular swarms in response to local tissue injuries or infection by microbes (Figure 1A). When neutrophils react to small laser-induced tissue damage in the mouse ear dermis, two-photon intravital microscopy (2P-IVM) allows the temporal resolution of distinct swarming phases (Figure 1B). Pioneer neutrophils switch from exploratory to chemotactic movement toward the tissue lesion, before they release signals to attract a second wave of neutrophils. In contrast to early swarming phases, the dynamics of crowding neutrophils during the late phase of swarming are more difficult to study by intravital imaging. Once neutrophils enter a growing cell cluster, delineating individual cells within the fluorescent mass of accumulating neutrophils is often challenging. Although genetic fluorescent labeling strategies allow, in principle, the identification of individual cells in dense neutrophil aggregates^6^ (Figure 1C), the resolution of focal 2P-IVM images from mouse tissue is commonly low and prevents more detailed biological insights. Moreover, the experimental access to neutrophil clusters in living mouse tissue is often restricted, preventing direct drug treatments or cell stainings. Given these technical limitations, the dissection of molecular mechanisms underlying neutrophil crowding *in situ* is experimentally challenging.

To overcome these limitations, we here build on a previously established *in vitro* neutrophil swarming assay, which has proven very valuable for studying human and mouse neutrophils.^9^^,^^11^^,^^15^^,^^24^ In the original experimental design of this assay, the biological particle clusters were patterned on standard microscope glass slides (thickness ∼1 mm), which allowed the observation of neutrophil swarm formation by bright-field and widefield fluorescence microscopy techniques. However, CLSM with standard 40×, 63×, and 100× objectives was not possible, as the thickness of the glass slide was incompatible with the working distances of common objectives. Therefore, we adapted our experimental design and utilized 22 mm × 70 mm coverslips with thickness 1 (0.13–0.16 mm) to manufacture circular patterns of heat-killed *S. aureus* (HKSA) BioParticles (Figure 1D). An additional adaptation that improved this platforms’ versatility was the ability to conjugate HKSA BioParticles to fluorophores (e.g., Alexa Fluor 647) that do not interfere with any wavelength utilized by the stains needed to visualize aspects of neutrophil structure or function. Moreover, we improved aspects of the device to avoid the drying of biological material, which could occur with the previous platform (see STAR methods). Importantly, our technical changes did not interfere with the swarming of mouse and human neutrophils and allowed the distinction of the sequential swarming phases: initiation, amplification, and crowding (Figure 1D and Video S1). As shown previously,^9^^,^^11^ neutrophil swarming in this swarming-on-a-chip platform depended on releasing the swarming factors LTB4 and CXCL2 (Figure S1A). Experiments in the adapted swarming-on-a-chip platform still showed that the extent of neutrophil swarming and accumulation scaled to differently sized micropatterns, as previously observed in the original experimental design.^9^ As expected, swarm sizes of both murine and human neutrophils scaled with printed HKSA patterns of 30 μm, 60 μm, and 120 μm diameter size (Figures S1B–S1D and Video S2).

Video S1. Swarming of mouse neutrophils in a swarming-on-a-chip platform, related to Figure 1D, upper row Lifeact-GFP-expressing mouse neutrophils (fluorescence intensity displayed as glow heatmap) swarm toward a 30-μm wide pattern of HKSA particles (cyan). Confocal fluorescence microscopy with 3.7 frames/min, projection of a z stack (8 slices, 6.26 μm). Time is displayed as min:s.

Video S2. Neutrophil crowding on differently sized HKSA patterns, related to Figure S1C Swarming of Lifeact-GFP-expressing mouse neutrophils (fluorescence intensity displayed as glow heatmap) on differentially sized HKSA pattern (30 μm, 60 μm, 120 μm). Pattern size is indicated with circles (cyan). Time is displayed as min:s.

Although these technical adaptations may sound simple and minor, they allowed us to gain unprecedented views into the dynamics of crowding neutrophils. When we combined live imaging with CLSM of Lifeact-GFP-expressing primary mouse neutrophils,^28^ we captured the complex morphology of dynamic multicellular neutrophil clusters. In particular, the acquisition of 3D stacks (*x-y-z*) allowed the post-imaging analysis of individual focal planes (*x-y*) or (*x-z*), where the outline of the actin cortex clearly demarcated individual cells in the neutrophil aggregate (Figure 1E and Video S3).

Video S3. Confocal 3D acquisition of crowding neutrophils, related to Figure 1E Lifeact-GFP-expressing mouse neutrophils (fluorescence intensity displayed as glow heatmap) swarm toward the HKSA pattern (cyan). Confocal microscopy with 1.4 frames/min. First part: projection of a z stack (16 slices, 9 μm). Second part: demonstration of *x-y* slicer (1.2 μm per section) and *y-z* slicer (1.23 μm per section). Time is displayed as min:s.

### Visualizing subcellular components in crowding neutrophils: mitochondria

**Figure 2:**
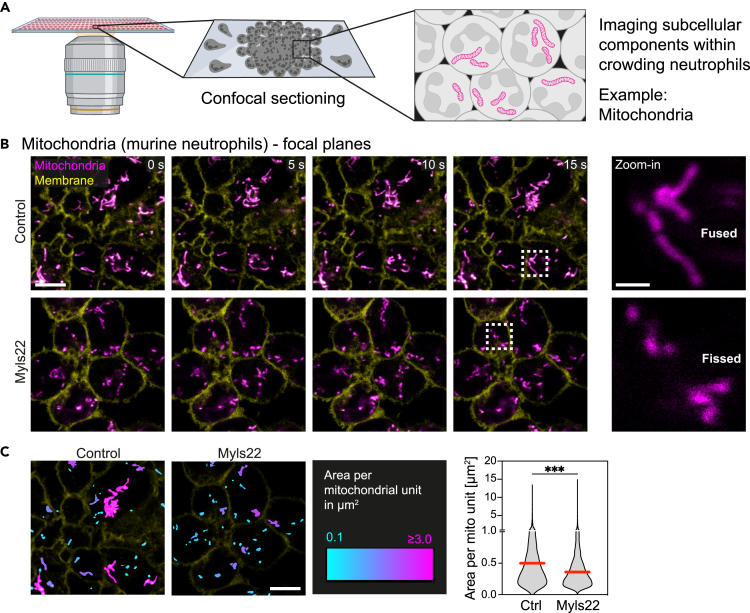
Visualizing subcellular components in crowding neutrophils, exemplified for mitochondria (A) Confocal imaging of swarming neutrophils allows to visualize and analyze subcellular structures such as mitochondria within swarm clusters. (B) Live-cell imaging of mitochondrial dynamics in individual cells of crowding mouse neutrophils over time. Mitochondria are stained with MitoTracker Green (magenta), and neutrophils express membrane-tagged tdTomato (yellow). Cells were untreated (upper row) or treated with the OPA1 inhibitor Myls22 (lower row). Zoom-in images compare elongated mitochondria in untreated neutrophils and fissed mitochondria in Myls22-treated neutrophils. Scale bar: 5 μm (time course) or 1 μm (zoom-in). (C) Post-imaging analysis of mitochondrial size (area per mitochondrial unit), which is color-coded in control or Myls22-treated mouse neutrophils (images from Figure 1G, 15s-timepoint). Scale bar: 5 μm (left). Quantification of mitochondrial size. Data from n = 3 independent experiments with in total *N* = 3678 (control) or 5182 (Myls22) mitochondrial units. Red bars indicate the median. Mann-Whitney test, ∗∗∗p < 0.0001. See also Figure S2 and Video S4.

Intravital imaging techniques and previous versions of the swarming-on-a-chip platform did not allow the imaging of swarming neutrophils at subcellular resolution, e.g., the visualization of intracellular organelles. Given their organelle size and the high quality of fluorescent reporter probes, mitochondria are often used as first-choice organelle when testing experimental imaging setups for their use for subcellular imaging. Mitochondria are key intracellular organelles with critical functions for cellular energy balance, calcium signaling, redox balances, and cell death.^29^ Studies from many different cell types have shown that mitochondria are highly dynamic organelles, which can change their intracellular position over a few minutes and adapt their morphology by coordinated cycles of fission and fusion.^30^ Hence, we asked whether we could use our adapted swarming-on-a-chip platform in combination with CLSM to visualize the morphologies and dynamics of mitochondria as an example for resolving subcellular structures in crowding neutrophils (Figure 2A). To test this, we imaged living neutrophils stained with MitoTracker dye, when they were crowding on HKSA patterns (Figure 2B and Video S4). We used primary mouse neutrophils that expressed membrane-tagged tdTomato to delineate single cells and assign mitochondria to individual neutrophils of the cell cluster. Microscopic analysis of confocal imaging planes clearly revealed elongated and fused shapes of mitochondria in mouse neutrophils (Figure 2B). Similarly elongated mitochondrial morphologies were observed in human neutrophils (Figure S2A). Thus, our imaging setup allowed the identification of fused mitochondrial states in crowding neutrophils. To further strengthen this point, we induced mitochondrial fragmentation in mouse neutrophils by treating cells with Myls22, an inhibitor of OPA1, the key regulator of inner mitochondrial membrane fusion.^30^^,^^31^ As expected, inhibition of OPA1-mediated mitochondrial fusion led to a fissed mitochondrial state, which we could microscopically resolve in our live-cell imaging setup (Figure 2B) and quantify by post-imaging analysis (Figure 2C). When treating neutrophils with a combination of a fusion promoter (M1) and a fission inhibitor (Mdivi-1),^32^ our results and quantitative analysis showed that the shape of mitochondria remained comparable to untreated control neutrophils (Figures S2B and S2C), supporting that mitochondria are in a maximally fused state during neutrophil crowding. Together, our data highlight the strength of the improved swarming-on-a-chip platform to visualize subcellular compartments in crowding neutrophils and rapidly identify subcellular processes, which could be worth further exploring.

Video S4. Mitochondria dynamics in crowding neutrophils, related to Figure 2B Mouse neutrophils expressing membrane-tagged tdTomato (yellow) are crowding on patterns of HKSA particles. Mitochondria are stained with MitoTracker Green (magenta). First part: Elongated mitochondrial morphologies in untreated cells. Second part: Neutrophils treated with OPA1 inhibitor Myls22 display fragmented mitochondrial morphologies. Confocal fluorescence microscopy with 12.7 frames/min (first part) or 12.1 frames/min (second part), single *z*-plane is displayed. Time is displayed as min:s.

### Visualizing functional perturbation of cell membrane and cortical actin integrity in crowding neutrophils

**Figure 3:**
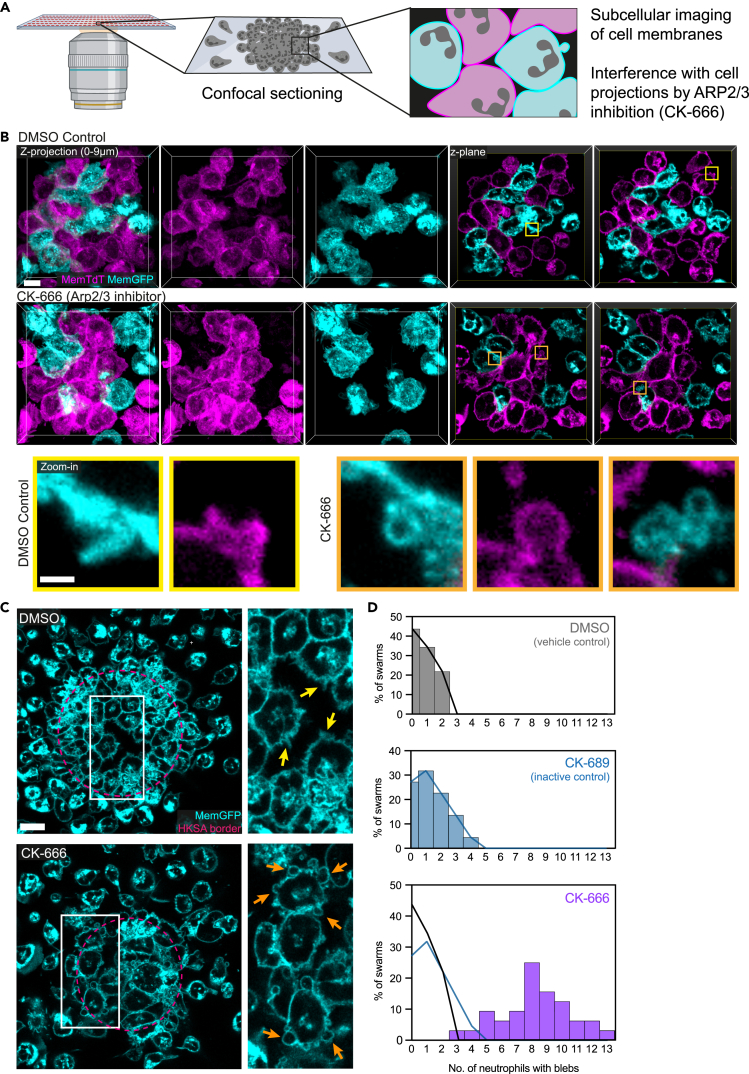
Arp2/3 complex inhibition causes membrane blebbing in neutrophil clusters (A) Confocal imaging of swarming neutrophils the detailed visualization of cell membranes in crowding neutrophils. (B) Mixed cell clusters of mouse neutrophils, which express either membrane-tagged GFP (cyan) or membrane-tagged tdTomato (TdT) (pink). Comparison of vehicle control (DMSO) with CK-666-treated neutrophils. Maximum intensity projection of a z stack (left) and single focal planes (right). Scale bar: 5 μm. Zoom-ins show filopodia-like structures in control neutrophils and membrane blebs in CK-666-treated neutrophils (lower row). Scale bar: 1 μm. (C) Focal planes of cell clusters formed by membrane-GFP-expressing mouse neutrophils (cyan), comparison of vehicle control with CK-666 treatment. The borders of HKSA patterns are indicated in red. Yellow arrows indicate filopodia-like structures, orange arrows indicate membrane blebs. Scale bar: 10 μm. (D) Quantification of the number of mouse neutrophils with membrane blebs per neutrophil swarm, comparison between DMSO (vehicle control)-, CK-689 (inactive control compound)-, and CK-666-treated neutrophils. Trend lines of DMSO and CK-689 are displayed in the CK-666 graph. Data from n = 3 independent experiments with *N* = 32 swarms (vehicle, CK-666) and *N* = 22 swarms (CK-689). See also Figure S3 and Video S5.

Having established that subcellular imaging of crowding neutrophils is feasible in our *in vitro* system, we next asked whether we could visualize details of cellular projections and membranes, when neutrophils dynamically interact inside cell clusters on 30 μm HKSA patterns (Figure 3A). We used primary mouse neutrophils that expressed membrane-tagged tdTomato or membrane-tagged GFP at a 1:1 ratio to identify cellular membranes of individual cells inside the clusters. Maximum fluorescence intensity projections of imaging datasets identified single cells, but cell borders were very difficult to delineate (Figure 3B, control). However, confocal sectioning and visualization of focal planes allowed a detailed analysis at cellular borders, showing that neutrophils form pseudopods in cell clusters (Figure 3B, zoom-in, control). Based on this observation, we asked about potential consequences of altering cellular protrusion dynamics in crowding neutrophils. To address this, we chemically interfered with the function of the Arp2/3 complex, a heptameric protein complex that is critical for the formation of branched actin networks and the integrity of the actin cortex at the interface between the cytoplasm and membrane.^33^ When we visualized crowding neutrophils, which were treated with the specific Arp2/3 complex inhibitor CK-666, by maximum intensity projections, we could not observe obvious differences to control neutrophil clusters (Figure 3B, CK-666). However, confocal sectioning identified the presence of small cellular blebs at the plasma membrane of crowding neutrophils (Figure 3B, zoom-in, CK-666). Experiments using primary mouse neutrophils with membrane-tagged GFP on 60 μm HKSA patterns and quantitative analysis confirmed these observations, demonstrating that CK-666 treatment increased the presence of membrane-blebbing neutrophils inside clusters in comparison to control conditions (Figures 3C and 3D). Importantly, we rarely detected membrane blebs in CK-666-treated neutrophils outside of HKSA patterns. Time-lapse microscopy experiments and confocal sectioning further supported that membrane bleb formation occurred mainly for neutrophils on HKSA patterns at later stages of neutrophil crowding (Figure S3A). While the early phases of blebbing are characterized by membrane detachment from the cortex and inflation, reassembly of the actin cortex under the membrane drives bleb retraction.^34^ When we used primary mouse neutrophils expressing Lifeact-GFP to visualize cortical actin, confocal sectioning revealed retracting blebs in CK-666-treated neutrophils on HKSA patterns, whereas neutrophils outside the patterns rarely displayed retracting blebs (Figure S3B). Thus, by using our adapted swarming-on-a-chip platform in combination with confocal sectioning, we identified cell membrane changes in crowding neutrophils, which we did not observe by widefield microscopy.

### Inhibition of Arp2/3 complex function causes death of crowding neutrophils

**Figure 4:**
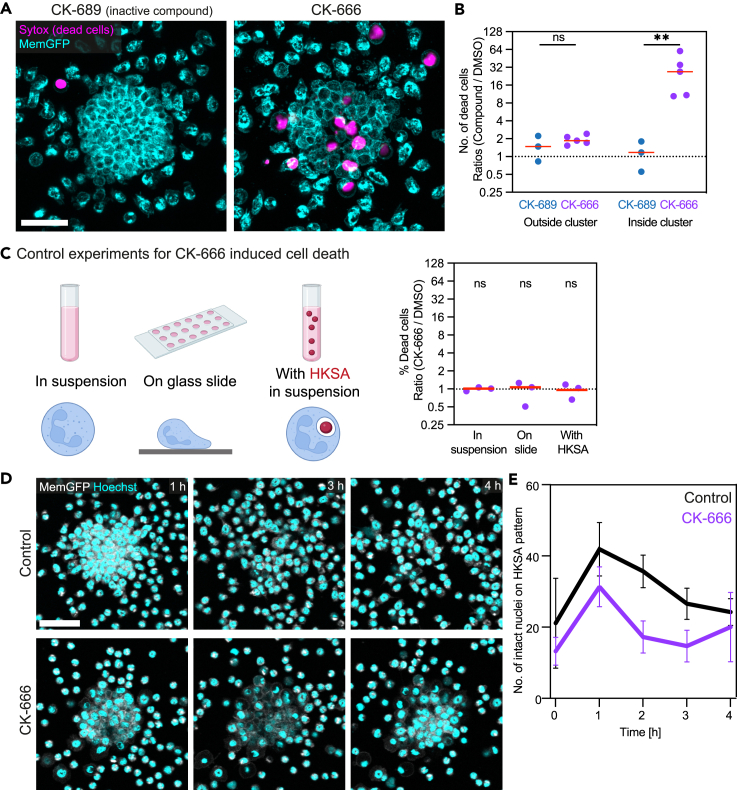
Arp2/3 complex inhibition causes cell death in neutrophil clusters (A) Visualization of cell death in swarms of CK-689- and CK-666-treated mouse neutrophils (cyan), stained with Sytox dye (magenta). Scale bar: 30 μm. (B) Quantification of cell death inside and outside of swarms per imaging field of view after 2 h. Analyzed are ratios of CK-689- or CK-666-treated neutrophils with DMSO-treated neutrophils. Data are from n = 3–5 independent experiments with *N* = 23–30 swarms each. Bars indicate the mean. t test of log2-transformed values, ns: not significant, ∗∗∗p < 0.001. (C) Effect of CK-666 treatment on neutrophil death in other experimental setups. Mouse neutrophils were incubated for 2 h in suspension, on glass slides or with HKSA in suspension (left). Quantification is based on n = 3 independent experiments. Ratios of CK-666- with DMSO-treated neutrophils were determined, bars indicate the mean. Data from n = 3 independent experiments. One sample t test, computed are log2-transformed values. ns: not significant (right). (D) Swarming of DMSO- and CK-666-treated memGFP (white)-expressing mouse neutrophils. Nuclei were stained with Hoechst (cyan). Scale bar: 40 μm. (E) Quantification of intact nuclei on HKSA patterns per swarm over 4 h of imaging, comparison between DMSO- and CK-666-treated mouse neutrophils. Data from n = 3 independent experiments with *N* = 3 swarms each. The graph displays the mean (solid line) with SD (error bars).

Blebbing is a common feature of cell physiology during cell movement, cytokinesis, cell spreading, and apoptosis.^35^ Based on our previous data that Arp2/3 complex inhibition caused bleb formation predominantly in crowding neutrophils on HKSA patterns, we hypothesized that these cells might undergo cell death. To address this, we performed *in vitro* swarming experiments with mouse neutrophils in the presence of the nucleic acid-staining Sytox dye, which does not pass membranes of living cells and thus indicates dead cells within a population. Indeed, inhibition of Arp2/3 complex function by CK-666 treatment resulted in a strong increase of Sytox-positive neutrophils inside neutrophil swarm clusters, which was not observed when cells were treated by CK-689, an inactive control compound (Figures 4A and 4B). This difference was not observed for neutrophils outside of neutrophil swarm clusters (Figure 4B). Importantly, CK-666 treatment did not induce the same increase in cell death, when neutrophils (i) were maintained non-adherent in suspension, (ii) were kept low-adherent on glass slides, or (iii) were phagocytosing HKSA bioparticles in suspension culture (Figure 4C). Thus, Arp2/3 complex inhibition caused neutrophil death under conditions, when crowding neutrophils were phagocytosing bioparticles from HKSA patterns. As a consequence, the dynamic pattern of neutrophil swarming was altered in the presence of CK-666 treatment. Control neutrophils rapidly accumulated and formed prominent neutrophil clusters within 1 h, before neutrophils left again and cell numbers declined in clusters over the following hours (Figure 4D). While CK-666 treatment still showed clear neutrophil recruitment and accumulation on HKSA patterns in the first hour, the occurring cell death caused a rapid decline of intact neutrophils over the next 2 h (Figure 4D). In agreement with previous findings where neutrophil death triggered swarm re-compaction *in situ*,^4^ we also observed secondary waves of intact neutrophils from peripheral regions, which raised the numbers of accumulating neutrophils again at later time points (Figure 4D and Video S5). Together, by following up observations from confocal sectioning of altered membrane and actin dynamics, our experiments identified an unexpected role of the Arp2/3 complex for cell survival in crowding neutrophils and neutrophil swarming dynamics.

Video S5. Swarming of CK-666 treated neutrophils, related to Figure 4D Swarming of membrane-tagged GFP (white)-expressing mouse neutrophils that were stained with Hoechst (cyan). Confocal microscopy with 0.13 frames/min. Time is displayed as h:min.

### Visualizing particle recognition and uptake during neutrophil crowding

**Figure 5:**
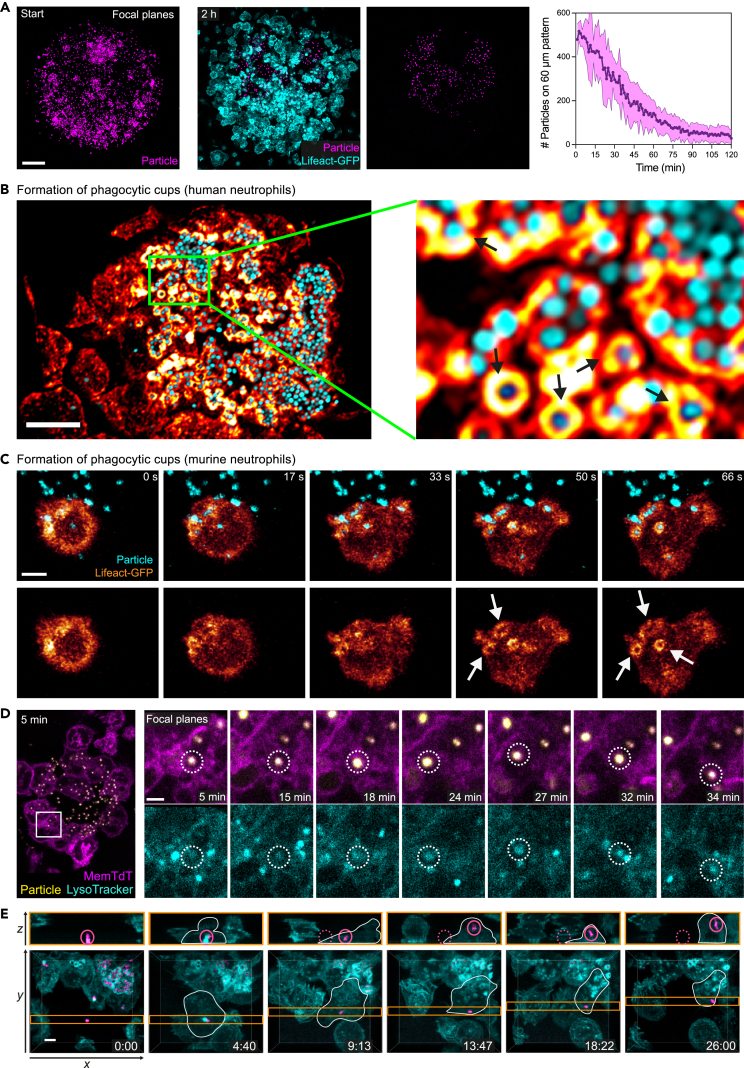
Visualizing particle recognition and uptake during neutrophil crowding (A) Comparison of HKSA patterns (magenta) before (left) and 2 h after the addition of Lifeact-GFP-expressing mouse neutrophils (cyan) (right). Scale bar: 20 μm. Quantitative analysis of particle decline on the coverslip over time. *N* = 5 swarms. Data points display the mean (solid line) with SD (shade). (B) Dye labeling of F-actin (glow) in fixed human neutrophils crowding on a pattern of HKSA particles (cyan). Black arrows in the zoom-in region show actin rings around bacterial particles. Scale bar: 10 μm. (C) Time series of an individual Lifeact-GFP (glow heatmap)-expressing mouse neutrophil, which forms phagocytic cups (white arrows) upon recognition of HKSA particles (cyan). Confocal z stacks are shown as projections. Scale bar: 3 μm. (D) Left: Overview of crowding mouse neutrophils (magenta, expressing membrane-tagged tdTomato) that take up HKSA particles (yellow). Right: The zoom-in shows the time course of one engulfed HKSA particle (dotted circle), which enters the phagolysosomal system, visualized by LysoTracker (cyan). Scale bar: 2 μm. (E) Visualization of a mouse neutrophil (cyan, cell borders outlined in white), which takes up a particle (magenta) and carries it away. Big pictures show confocal *z* stacks as projections. *Z*-slices at the position of the particle (orange-lined stripe) are displayed in the upper row. Position of the particle (full magenta circle) and original position of the particle (dotted magenta circle) are indicated. Scale bar: 3 μm. See also Video S6.

When neutrophil populations start to form dense cell clusters, they concentrate the bactericidal activity of many individual neutrophils into one spot. How neutrophil clusters realize the uptake and elimination of microbes has not yet been studied in detail. In our experimental setup we observed that the number of surface-bound HKSA particles decreased over time, suggesting that crowding neutrophils eliminated these particles (Figure 5A). Confocal sectioning allowed a close examination of the filamentous actin (F-actin) distribution in crowding neutrophils, revealing ring-like actin structures around fluorescent particles reminiscent of phagocytic cups (Figure 5B and Video S6). Live confocal imaging of individual Lifeact-GFP-expressing mouse neutrophils and visualization of confocal planes showed the rapid formation of actin-based phagocytic cups only seconds after the cells had touched the HKSA particles (Figure 5C). When living neutrophils were loaded with LysoTracker dye, we could spatiotemporally resolve how individual particles (i) were taken up by the phagosome, (ii) moved within the cell (Figure 5D, 5–24 min), and (iii) became acidified over time in the phagolysosome (Figure 5D, 24–34 min). Furthermore, acquiring 3D stacks over time often also allowed fate tracking of the engulfed particle. As an example, Figure 5E and Video S4 display a neutrophil that was attracted to a growing neutrophil cluster and encountered a single HKSA particle. Actin concentrated at the site of the particle (Figure 5E, 4:40), and, 5 min later, the particle was ripped away from the surface and then taken up into the cell (Figure 5E, 9:13) before it was carried away by the neutrophil (Figure 5E, 13:47–26:00). Thus, confocal sectioning in our imaging platform is also feasible to visualize particle recognition and uptake in single cells inside multicellular neutrophil clusters. Building on the microscopic identification of these early steps of neutrophil activation, we next investigated the spatiotemporal induction of effector functions in developing neutrophil clusters.

Video S6. Particle uptake and removal by individual neutrophils of the swarm, related to Figure 5E Swarming Lifeact-GFP-expressing mouse neutrophils (cyan) take up and carry away HKSA particles (magenta). Confocal fluorescence microscopy with 0.67 frames/min, projection of a *z* stack (21 slices, 7.4 μm). Time is displayed as min:s.

### Visualizing spatiotemporal patterns of ROS production in developing neutrophil swarms

**Figure 6:**
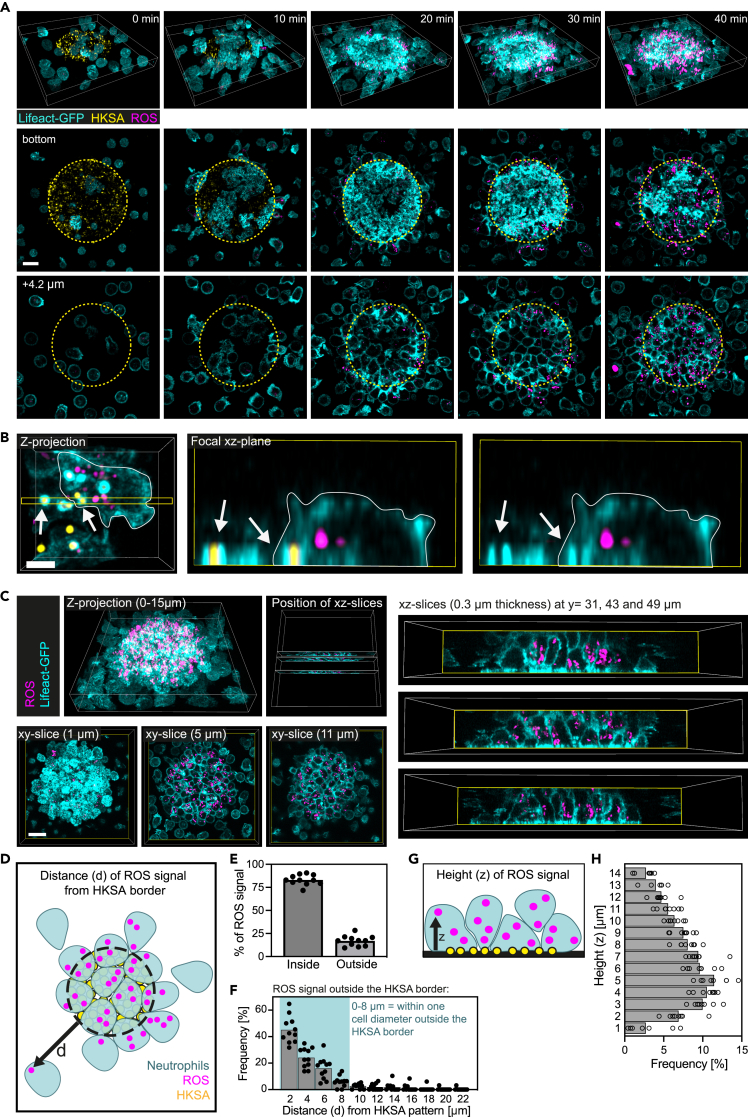
Visualizing spatiotemporal patterns of ROS production in developing neutrophil swarms (A) ROS production within developing swarms of mouse neutrophils over 40 min. Lifeact-GFP (cyan)-expressing mouse neutrophils were loaded with BioTracker Orange OH and HClO dye (magenta). Focal planes of 1.8 μm at the bottom (patterned HKSA, yellow) and 4.2 μm above the bottom plane. Scale bar: 10 μm. (B) Confocal imaging of ROS (magenta) localization within a mouse neutrophil (Lifeact-GFP, cyan) in comparison to HKSA (yellow, indicated by the arrows). Scale bar: 4 μm. (C) Confocal imaging of Lifeact-GFP-expressing mouse neutrophils (cyan) allows precise localization of ROS signals (magenta) in all three dimensions. Scale bar: 15 μm. (D) Analysis of ROS signal localization in *x-y* dimension based on measurements of the distance d from the HKSA border. (E) Quantification of the *x-y* localization of ROS signal. Frequency of the number of distinct ROS signals inside versus outside of the swarm. Data from *N* = 11 swarms, with *N* = 140–298 ROS units per swarm. (F) Localization of the ROS signal outside the swarm. Most ROS outside the HKSA border is produced within a distance d of one cell diameter (∼8 μm). Data from *N* = 11 swarms, with *N* = 24–82 ROS units outside the HKSA border. (G) The localization of the ROS signal in *z* is measured as the height *z* from the focal plane containing the HKSA particles. (H) Quantification of the height *z* of ROS signals in neutrophil swarms. Data from *N* = 8 swarms, with *N* = 577–991 ROS units per swarm. See also Figure S4 and Video S7.

In order to fulfill their function of killing bacteria and other microbes, neutrophils engulf pathogens and activate nicotinamide adenine dinucleotide phosphate (NADPH) oxidase to generate superoxide, which acts as the precursor for ROS.^36^ Previous work has shown that the activity of NADPH oxidase and ROS signaling are dispensable for initiating neutrophil swarming.^13^^,^^16^^,^^18^ Based on the use of neutrophils from NADPH oxidase-deficient chronic granulomatous disease (CGD) patients in these studies, ROS production appears to emerge as a control mechanism to avoid over-recruitment of neutrophils at late stages of swarming. However, none of these studies have pinpointed the sources of ROS production in populations of swarming neutrophils. Recent work proposed ROS-mediated negative feedback control models of neutrophil swarming without directly visualizing ROS signals in developing neutrophil swarms.^16^ By using a commercially available live-cell imaging probe to detect hydroxyl radicals and hypochlorous acid in living Lifeact-GFP-expressing neutrophils, we asked whether we could use our imaging setup and confocal sectioning for the visualization of ROS production in swarming neutrophils. Visualization of phagocytic cup formation around patterned HKSA particles at the bottom focal plane allowed us to clearly determine the contact of neutrophils with the neutrophil activation signal (Figures 6A and 6B). Already 15–20 min after the first contact of individual neutrophils with HKSA particles, fluorescent ROS signals were visible as vesicular pattern, mostly in confocal sections above the bottom focal plane. These data indicated that ROS production started in the phagosomes of early-arriving neutrophils (Figures 6A, 6B, and Video S7). Next, we used our confocal imaging datasets for a detailed spatial analysis of ROS activation in established neutrophil clusters. After 40 min, the majority of ROS signal (∼80%) in the neutrophil population was restricted to cells crowding on the HKSA pattern (Figures 6A and 6C–6E). Detailed analysis of *x-y* focal planes showed that the remaining ROS signals (∼20%) were found in neutrophils located within one cell diameter (0–8 μm) outside the HKSA border (Figures 6E and 6F). Analysis of *x-y* focal planes also confirmed that ROS-related signals can be detected away from the HKSA-patterned focal plane, mainly within a distance that corresponds to one cell diameter (*x-z*: 1–8 μm) (Figures 6G and 6H). Thus, ROS production remains largely restricted to crowding neutrophils, which receive their activation signal on HKSA patterns. ROS-related signals of activated neutrophils did commonly not extend further than one additional cell diameter from HKSA particles. Only in rare cases did we observe that individual neutrophils, which acquired ROS-related fluorescence signals after initial contact with the HKSA patterns, were pushed out from a cluster over time by other neutrophils (Figure S4). Together, our data show that neutrophil activation by HKSA particles induces neutrophil clustering and ROS production. Cellular ROS signals remain locally concentrated in neutrophil clusters rather than being propagated to individual cells outside clusters.

**Figure 7:**
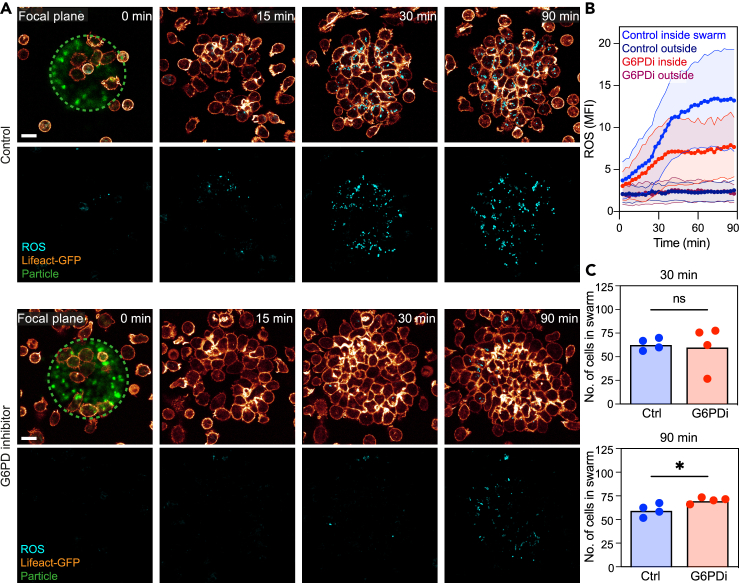
Pentose phosphate pathway controls ROS production in crowding neutrophils (A) Live-cell imaging of ROS (cyan) production in growing mouse neutrophil (Lifeact-GFP. glow) swarms over time. The dotted circle indicates the position of HKSA particles (green). Cells were untreated (upper two rows) or treated with G6PD1 inhibitor (lower two rows). Scale bar: 10 μm. (B) Quantitative analysis of ROS signals in mouse neutrophils over time. Compared are the area within the swarm and outside of the swarm, in untreated (blue) or G6PD1-inhibitor-treated (red) conditions. Data points are mean fluorescence intensity signals of the BioTracker dye. Data points display the mean (solid line) with SD (shade). *N* = 24 swarms per condition of n = 3 biological replicate. (C) Quantification of swarm size (mouse neutrophils) after 30 min and 90 min upon ROS inhibition with G6PDi. *t* test, ns: not significant, ∗p < 0.05.

Video S7. Visualization of ROS production in crowding neutrophils, related to Figure 7A Swarming of Lifeact-GFP-expressing mouse neutrophils (fluorescence intensity displayed as glow heatmap) that were stained with BioTracker Orange OH and HClO (cyan). Confocal microscopy with 0.41 frames/min, a single *z*-plane is displayed. Time is displayed as min:s.

### PPP controls ROS production in crowding neutrophils

Previous work has shown that ROS production depends critically on the availability of reduction equivalents like NADPH. Inhibition of the PPP, the main source of NADPH, was shown to efficiently inhibit the oxidative burst upon pathogen contact.^37^^,^^38^ Whether and how the PPP regulates aspects of neutrophil swarming has never been tested. To address this question, we treated cells with an inhibitor of glucose-6-phosphate dehydrogenase (G6PDi), the initial enzyme controlling the PPP. In agreement with the reported roles of the PPP for controlling NADPH oxidase activity, G6PDi treatment substantially decreased fluorescent ROS signals in crowding neutrophils (Figures 7A and 7B). Blocking G6PD activity did not affect the early phase (30 min) of neutrophil accumulation and cluster growth (Figure 7C). However, neutrophil cluster sizes increased upon G6PDi treatment at later phases of the swarming response (90 min) (Figure 7C). Thus, metabolic interference with the PPP impairs ROS production in crowding neutrophils and leads to significantly increased neutrophil cluster growth over time.

## Discussion

Neutrophil swarm formation shows striking parallels to the defensive behavior of Asian honeybees, which form large clusters of densely accumulating individuals, termed “thermoballs,” when defending their bee hive from intruding hornets.^39^ Similar to the Asian honeybees that isolate hornets from the healthy hive and kill the predators by thermoballing, neutrophils form cell aggregates to isolate local sites of infection from healthy tissue and eliminate microbial invaders. The development and maintenance of a defensive multicellular cluster appear very complex, and it is currently unknown whether and how individual cells coordinate their functions to realize a combined protective response. It appears likely that individual cell responses, including the phagocytosis of microbes, the cellular activation and metabolic state, the release of anti-microbial compounds, the secretion of cytokines, and the interaction with neighboring cells are heterogeneously organized inside a multicellular cluster of neutrophils. Strikingly, we have only little knowledge about the dynamic regulation and organization of the neutrophil crowding response.

Although cell crowding is a general phenomenon of many collective cell behaviors,^40^^,^^41^ the biological aspect of neutrophil crowding, when many neutrophils concentrate locally and compete for space and nutrients, has been largely neglected. This is mainly due to a missing methodology for imaging single-cell dynamics in dense neutrophil clusters in real time. In principle, live imaging of neutrophil crowding faces similar challenges to imaging cell dynamics of other collectively migrating cell systems, e.g., invading strands of cancer cells,^42^ epithelial cell monolayers,^43^ *Drosophila* primordial germ cells,^44^ zebrafish lateral line primordium,^45^ clusters of malignant lymphocytes,^46^ or organoids.^47^ This mainly refers to the problem of discriminating individual cells in dynamic cell aggregates. Compared to other cell collectives, individual cells in swarming neutrophil populations have high migration speeds, which pose an additional challenge to live-cell imaging experiments.

We here built on previous versions of a swarming-on-a-chip platform and improved it for the use with CLSM, which has previously not been applied to the study of subcellular dynamics in swarming neutrophils. Confocal sectioning allowed us to visualize subcellular components and processes, which could not have been resolved or dynamically imaged by previously published *in vitro* setups. As an example, we here show that the visualization of mitochondria inside neutrophil clusters is possible in our experimental setup, and that crowding neutrophils display fused and elongated mitochondria. By combining chemical inhibitors with the live visualization of mitochondria, we rapidly identified that the mitochondrial fusion program appears constantly active in crowding neutrophils, an unexpected finding that warrants the time-consuming and costly establishment of genetic models and further studies in inflammatory tissue settings. This example alone already shows the great potential of swarming-on-a-chip platforms for the rapid identification of biological processes, which may be worth further examination.

Moreover, CLSM and confocal sectioning allowed us to identify CK-666-induced alterations in cell membrane and cortical actin integrity, which we did not detect in maximum intensity projections that correspond to standard non-confocal imaging datasets. To our surprise, inhibition of the Arp2/3 complex specifically caused cellular bleb formation of neutrophils crowding on HKSA patterns. Given its roles for nucleating the formation of actin filaments and branched actin networks underneath the plasma membrane,^33^ the Arp2/3 complex has important functions in many immune cell types. However, the phenotypic consequences of interfering with Arp2/3 complex function depend on the studied immune cell type. While Arp2/3 complex blockade of dendritic cells and macrophages results in switches from lamellopodial to filopodial motility,^48^^,^^49^ partial loss of Arp2/3 complex function in T cells leads to bleb-based migration.^50^ Actin-based pseudopods of neutrophil-like dHL60 cells show intrinsically lamellar organization, which can be switched to filopodial organization by CK-666 treatment.^51^ Interestingly, blockade of Arp2/3 complex function in dHL60 cells leads to the formation of bleb-based protrusions under confinement.^52^ Our experiments with primary mouse neutrophils showed that CK-666-induced bleb formation coincided with the death of neutrophils crowding on HKSA patterns. Since HKSA stimulation of CK-666-treated neutrophils in suspension did not show this phenotype, we speculate that activated neutrophils in cell clusters experience additional factors, e.g., pushing and pulling forces through neighboring cells, which might promote cell death under conditions of a weakened actin cortex. Future experimentation will need to address these questions.

We also used CLSM to visualize and pinpoint the source of ROS production in neutrophil swarms. By using a commercially available fluorescent probe for detecting ROS species, we show that ROS production is largely restricted to neutrophil clusters on HKSA patterns and rarely detected in individual neutrophils outside of clusters. Since high levels of intracellular calcium precede ROS production,^47^ it is interesting to compare our results to a very recent study, which studied calcium waves in swarming neutrophils.^16^ This work showed that the initial calcium influx of neutrophils, which accumulate on micropatterned fungal particles, is followed by multiple waves of calcium activity that radially propagate away from the center to surrounding neutrophils. Rising levels of intracellular calcium also stimulate the synthesis and release of the swarm attractant LTB4,^5^ which diffuses from central neutrophil clusters, induces transient calcium spikes in surrounding neutrophils, and thus leads to the radial propagation of calcium waves.^27^ Based on this, an NADPH oxidase-based negative feedback loop has been proposed to self-extinguish the LTB4-driven propagation of calcium waves and prevent over-recruitment of neutrophils.^16^ Since we detected ROS signals predominantly in the central areas of neutrophil swarms, our data suggest that this negative feedback loop might exert its function from centrally crowding neutrophils.

Moreover, our data further show that the NADPH oxidase-based negative feedback loop is under metabolic control. Using chemical inhibitor treatment, we show that the PPP, an anabolic metabolic pathway that acts in parallel to glycolysis, controls ROS production of centrally clustering neutrophils in contact with bacterial particles. While the PPP is dispensable for the early phases of neutrophil swarming and the onset of neutrophil crowding, this metabolic pathway has a functional role in terminating neutrophil cluster growth. These findings are in agreement with the current view that NADPH oxidase activity acts as a swarming terminator.^16^ A role of PPP-regulated formation for NADPH oxidase-dependent neutrophil extracellular trap (NET) formation^38^ can be excluded, as we do not observe NETosis in our assay system with heat-killed bacterial particles. Thus, metabolic interference with the PPP appears to specifically impair the anti-microbial capacity of a neutrophil swarm at the cost of impaired neutrophil swarm termination.

We here expanded the use of swarming-in-a-chip platforms to acquire 3D stacks (*x-y-z*) or individual focal *z*-planes (*x-y*) by standard CLSM. However, this experimental platform has the potential to be used with other more advanced microscopy techniques. Line scanning during standard confocal microscopy limits the acquisition time of 3D stacks and prevents the capture of fast neutrophil processes, e.g., cell protrusion dynamics. Spinning-disk confocal and lattice light-sheet microscopy are likely to expand the detailed spatiotemporal information on 3D cellular shapes and motion,^53^ which can be studied in populations of crowding neutrophils.

### Limitations of the study

The here-described assay allows the visualization of subcellular aspects of neutrophil crowding, which have so far been challenging to study *in vivo*. While this expands the range of imaging applications for studies on neutrophil swarming dynamics, we can imagine further technical improvements to mimic the physiological situation even better. The current device supports neutrophil swarming on a 2D glass surface, but we did not achieve yet an experimental system to study neutrophil swarming in a truly 3D environment, which would likely support the formation of more spherical neutrophil aggregates. However, all *in vitro* experimental platforms cannot fully recapitulate the complexity of mammalian tissues, particularly during inflammatory and other pathophysiological conditions. Neutrophils are studied without their physiological context, including intact organ structure and interactions with other immune and non-immune cells. This also extends to other physiological parameters, such as lymph flow or nutrient availability, which are not fully recapitulated in *in vitro* systems. Thus, a combination of *in vitro* and *in vivo* methods should be considered for future studies of mammalian neutrophil swarming.

## STAR★Methods

### Key resources table

**Table:** 

REAGENT or RESOURCE	SOURCE	IDENTIFIER
**Antibodies**		

β-Tubulin (9F3) Rabbit mAb	Cell Signaling Technology	Cat#2128; RRID: AB_823664
Anti-rabbit IgG (H + L), F(ab')_2_ Fragment (Alexa Fluor® 488 Conjugate)	Cell Signaling Technology	Cat#4412; RRID: AB_1904025

**Chemicals, peptides, and recombinant proteins**		

LysoTracker DeepRed	Invitrogen	Cat#L12492
MitoTracker Green	Invitrogen	Cat#M7514
BioTracker Orange OH and HClO	Sigma-Aldrich	Cat#SCT038
CellTracker Green	Invitrogen	Cat#C34552
Myls22	MedChemExpress	Cat#HY-136446; CAS: 306959-01-3
G6PDi-1	Sigma-Aldrich	Cat#SML2980; CAS: 2457232-14-1
SB225002	Tocris	Cat#2725; CAS: 182498-32-4
MK-886	Abcam	Cat#141140; CAS: 118414-82-7
Mdivi-1	Sigma-Aldrich	Cat#M0199-5MG; CAS: 338967-87-6
Mitochondrial fusion promoter M1	Sigma-Aldrich	Cat#SML0629-25MG; CAS: 219315-22-7
Hoechst 33342	Thermo Fisher Scientific	Cat#62249
Sytox™ Orange nucleic acid stain	Thermo Fisher Scientific	Cat#S11368
CK-666	Merck Millipore	Cat#182515; CAS: 442633-00-3
CK-689	Merck Millipore	Cat#182517; CAS: 170930-46-8
MitoTracker™ Red FM	ThermoFisher Scientific	Cat#M22425
BioTacker™ 488 Green Microtubule Cytoskeleton Dye	Sigma-Aldrich	Cat#SCT142
SPY650-FastAct™	Cytoskeleton, Inc.	Cat#CY-SC505
DAPI (4′,6-Diamidino-2-Phenylindole, Dilactate)	Life Technologies	Cat#D3571
ActinRed™ 555 ReadyProbes™ Reagent (Rhodamine phalloidin)	ThermoFisher Scientific	Cat#R37112
ProLong™ Glass Antifade Mountant	ThermoFisher Scientific	Cat#P36982
Alexa Fluor™ 647 NHS Ester (Succinimidyl Ester)	Invitrogen	Cat#A20006
DC 184 Sylgard Kit	Krayden	Cat#DC4019862
Zetag 8185	BASF	Cat#056187901PCDF
SU8-2050	Microchem	Cat#Y111072-1GL
SU8 Developer	Microchem	Cat#Y020100 4000L1PE
*Staphylococcus aureus* (Wood strain without protein A) BioParticles™, unlabeled	Invitrogen	Cat#S2859
*Staphylococcus aureus* (Wood strain without protein A) BioParticles™, Alexa Fluor™ 488 conjugate	Invitrogen	Cat#23371

**Critical commercial assays**		

Neutrophil Isolation Kit, mouse	Miltenyi Biotec	Cat#130-097-658
EasySep™ Human Neutrophil Isolation Kit	StemCell Technologies	Cat#17957

**Experimental models: Organisms/strains**		

Mouse: Tg(Lifeact-GFP): Tg(CAG-EGFP)#Rows	Roland Wedlich-Söldner	MGI: 4831036
Mouse: Membrane tdTomato: ROSA^mT/mG^	The Jackson Laboratory	JAX 007676
Mouse: Commd10^Tg(Vav1−icre)^	The Jackson Laboratory	JAX 008610
Mouse: Membrane GFP: Tg(Vav1-Cre) ROSA^mT/mG^	MPI Freiburg	
Mouse: C57BL/6J-Albino: Tyr^c−2J^/^c−2J^	The Jackson Laboratory	JAX 000058
Mouse: Tg (dsRed): Tg(CAG-DsRed∗MST)1Nagy	The Jackson Laboratory	JAX 006051
Mouse: Tg(dsRed) Tyr^c−2J^/^c−2J^	MPI Freiburg	

**Software and algorithms**		

Imaris (version 9.5)	Bitplane	https://imaris.oxinst.com
Fiji/ImageJ2 (version 2.9.0)	Schindelin et al. (ref. 49)	https://imagej.net/ij/index.html; https://fiji.sc
Nikon NHS-Elements	Nikon, Tokyo, Japan	AR, C, Viewer
FluoView	Olympus, Tokyo, Japan	Olympus FW31S-SW
JMP	JMP Statistical Discovery, North Carolina, United States	N/A
ZEN (black edition)	Carl Zeiss Microscopy	https://www.micro-shop.zeiss.com/de/de/softwarefinder/software-categories/zen-black/#
AIM (version 4.0)	Carl Zeiss Microscopy	N/A

### Resource availability

#### Lead contact

Further information and requests for resources and reagents should be directed to and will be fulfilled by the lead contact, Tim Lämmermann (laemmermann@ie-freiburg.mpg.de).

#### Materials availability

This study did not generate new unique reagents.

#### Data and code availability

• All data reported in this paper will be shared by the lead contact upon request.
• This paper does not report original code.
• Any additional information required to reanalyze the data reported in this paper is available from the lead contact upon request.

### Experimental model and subject details

#### Mouse models

Mice were bred and maintained in a conventional animal facility at the Max Planck Institute for Immunobiology and Epigenetics or in a specific pathogen-free animal facility at the Association for Assessment and Accreditation of Laboratory Animal Care-accredited animal facility at the National Institute of Allergy and Infectious Diseases (NIAID) according to local regulations. Animal breeding and husbandry was conducted with approval by the local authorities. Animal breeding and husbandry at the Max Planck Institute were performed following the guidelines provided by the Federation of European Laboratory Animal Science Association and by German authorities and the Regional Council of Freiburg. Intravital imaging procedures were done according to a study protocol approved by the NIAID Animal Care and Use Committee (Bethesda, USA). All mouse strains used in this study were without health burdens. Adult mice (>8 weeks) of both sexes were used. Mouse strains used in this study have been described: C57BL/6J (Jax strain No. 000664), *Tyr*^*c−2J*^*/J* (Jax strain No. 000058), *Tg(Lifeact-GFP)* (MGI: 4831036, kindly provided by Roland Wedlich-Söldner (University of Münster, Germany)),^28^ *ROSA*^*mT/mG*^ (Jax strain No. 007676),^54^ *Commd10*^*Tg(Vav1-icre)*^ (Jax strain No. 008610).^55^

#### Human blood

Human blood was collected according to protocol #2018H0268 approved by the Biomedical Sciences Committee Institutional Review Board (IRB) at The Ohio State University.

### Methods details

#### Bioparticle device preparation

An established photolithography technique was used to fabricate a polydimethylsiloxane (PDMS) microstamp outlined in Walters et al.^14^ Concisely, SU-8 2050 (Kayaku Advanced Materials, Westborough, MA) was spun to a 40-μm thick layer on a silicon wafer (University Wafers, South Boston, MA). Subsequently, this wafer was exposed to UV light through a chrome mask that was constructed to the desired pattern, arrays of either 30 μm, 60 μm, or 120 μm in diameter circles and 150 μm, 400 μm, 400 μm center to center spacing, respectively. The UV light crosslinked the SU-8, which maintained the pattern within the mask. The remaining SU-8 was then removed with photoresist developer to leave the developed pattern on the wafer. The wafer was then incubated with a 10:1 ratio of PDMS and its curing agent (Dow, Midland, MI), then allowed to degas in a vacuum to allow for the PDMS to fill into the pattern. Once the air bubbles were visibly gone, the PDMS full wafer was incubated at 65°C overnight. The cured PDMS was then cut out of the wafer, resulting in the completed microstamps.

The bioparticle microarray was produced through a microstamping procedure established in Walters et al.^14^ and adapted for the use of 22 × 70–1 coverslips (Fisher Scientific, Pittsburg, PA). Concisely, 200 μL of Zetag 8185 (1.6 mg/mL, BASF, Ludwigshafen, Germany) was spun onto a clean glass slide, and the microstamp with the desired array was allowed to incubate on the Zetag layer for 20 min, under a pressure of 3.8 g/cm^2^, to equally distribute Zetag onto the microstamp. Once this time has elapsed, the microstamp was transferred to a coverslip that was cleaned through three 3-min ultrasonication (Fisher Scientific, Pittsburg, PA) cycles between DI water and 70% ethanol, followed by a 5-min exposure to ozone (Jelight Company Inc., Irvine, CA), and then a 1-min exposure to oxygen plasma (Plasma Etch, Carson City, NV). The microstamps were applied the same pressure (3.8 g/cm^2^), removed after a 10-min incubation, and the newly formed Zetag array was dried at room temperature overnight. An 8-well silicon mold (Grace Bio-Labs, Bend, OR) was placed onto the stamped coverslips and 50 μL of *Staphylococcus aureus* (67 μg/mL, Wood strain without protein A) bioparticles conjugated to AlexaFluor 647 (ThermoFisher, Waltham, MA) were added to each well and allowed to incubate for 2 h at room temperature. This caused the bioparticles to congregate around the microstamped Zetag array through electrostatic force. The excess bioparticles were subsequentially washed away under a stream of DI water. Thus, the array was produced, with the bioparticles acting as targets for neutrophil crowding. The bioparticle microarrays can be stored for 3 months at 4°C sealed from outside interactions.

#### Human neutrophil isolation

Human blood was collected the morning of an experiment in K2-EDTA tubes (BD Vacutainer, Fisher Scientific). The freshly collected blood was then added at a 1:20 ratio to a cell lysis buffer of 150 mM ammonium chloride, 10 mM sodium bicarbonate, and 0.1 mM EDTA at 7.4 pH for 5 min. This solution was then centrifuged at 350 × g for 5 min to isolate the leukocytes. All leukocytes, except for neutrophils, were removed with the EasySep Human Neutrophil Isolation Kit (STEMCELL Technologies, Vancouver, Canada), which isolated the neutrophils through negative selection via immunomagnetic separation. Neutrophils were resuspended in Iscove’s Modified Dulbecco’s Medium (IMDM, ThermoFisher), and stained with 1× FastAct actin probe (SpiroChrome) for 1 h and 20 μg/mL Hoechst 33342 (ThermoFisher) for 10 min. To wash the stained neutrophils, PBS was added to 5-times the original cell staining volume, centrifuged at 1000 revolutions per minute (RPM), and the supernatant was aspirated. The neutrophils were finally resuspended in IMDM, with 20% FBS (Gibco, Thermofisher) and 1% penicillin-streptomycin (PS, Gibco, Thermofisher) at a concentration of 1.2 × 10^6^ cells/mL.

#### Human neutrophil immunofluorescence

Neutrophils were allowed to crowd on the bacterial microarray for 60 min prior to fixation and permeabilization. The neutrophils were fixed with 4% formaldehyde (Cell Signaling Technology, Danvers, MA) in PBS for 15 min. The fixed cells were then permeabilized with 0.1% Triton X-100 (Sigma-Aldrich, St. Louis, MO) for 10 min. The permeabilized cells were then blocked with 10% normal goat serum for 1 h, followed by an incubation with a primary anti-β-tubulin antibody (2128; Cell Signaling Technology, Danvers, MA) at a dilution of 1:100 in 1% bovine serum albumin (BSA; Sigma-Aldrich) in 0.07% Tween 20 (Sigma-Aldrich). After an overnight incubation of the primary antibody at 4°C, secondary antibodies were incubated at a 1:1000 dilution in 1% BSA in 0.07% Tween 20. The actin filaments of the neutrophils were later stained with ActinRed 555 ReadyProbes reagent, according to the manufacturer’s protocol. Lastly, the nuclei were stained with a 300 nM 4′,6-diamidino-2-phenylindole (DAPI; Thermo Fisher Scientific, Waltham, MA) solution in PBS for 5 min. The stained cells were mounted with ProLong Glass Antifade Mountant (Thermo Fisher Scientific) and imaged via epifluorescence (Nikon, Melville, NY) and confocal (Olympus, Tokyo, Japan) microscopy. Washing steps were performed three times between all incubations (except after blocking) with PBS for 5 min.

#### Isolation, staining and treatment of murine neutrophils

Bones (tibia, femur and os coxae) were dissected, sterilized in ethanol and gently crushed using pestle and mortar in cold wash buffer (HBSS, 2% FBS, 2 mM EDTA). Cell suspension was filtered through a 70 μm strainer and pelleted at 300 × g for 5 min. Erythrocyte lysis was performed with 1 mL ACK lysis buffer (Gibco) and stopped after 1 min with buffer. For *in vitro* experiments, neutrophils were isolated from the bone marrow cell suspension using the MACS neutrophil isolation kit for negative selection (Miltenyi Biotec) and an autoMACS ProSelector cell separator (Miltenyi Biotec) according to the manufacturer’s protocol. Isolated murine neutrophils were resuspended at a concentration of 5 × 10^6^ cells/mL in RPMI, supplemented with 10% FBS and 2 mM glutamin, and kept on ice. Lifeact-GFP expressing neutrophils were isolated from transgenic Lifeact-GFP mice. Neutrophils with membrane-tagged tdTomato were isolated from *ROSA*^*mT/mG*^ mice. Neutrophils with membrane-tagged GFP were isolated from *Vav1-iCre*^*+/−*^ *ROSA*^*mT/mG*^ mice. For injection experiments, neutrophils from bone marrow of C57BL/6J mice were isolated using a Percoll gradient (78%, 69% and 52%) and afterward washed three times in wash buffer. Then, they were dye-labeled with 1 μM CellTracker Green (Invitrogen) in HBSS with 0.0002% pluronic F-127 (Thermo Fisher Scientific). Neutrophils were washed four times and then resuspended at a concentration of 2 × 10^6^ cells/mL in PBS. To visualize lysosomes or mitochondria in *in vitro* experiments, neutrophils were stained with 25 nM LysoTracker DeepRed or 150 nM MitoTracker Green (both Invitrogen) respectively for 30 min at 37°C and 5% CO_2_ in a humidified incubator and washed once before imaging. ROS was visualized using 1 μM BioTracker Orange OH and HClO Live cell dye (Sigma-Aldrich). To visualize nuclei, neutrophils were stained with Hoechst (Thermo Fisher Scientific). To assess cell death in the swarming assay, neutrophils were stained with Sytox Orange (Thermo Fisher Scientific). To quantify cell death in control experiments, neutrophils were incubated in tubes, on glass slides or with opsonized HKSA (woodstrain without protein A, invitrogen) at a ratio of 1:100 for 2 h at 37°C and 5% CO_2_ in a humidified incubator. For experiments with neutrophils in solution, DAPI was added and cell death assessed using a LSRFortessa flow cytometer. For experiments with neutrophils on glass slides, staining with Sytox Orange and microscopical analyses were used.

The inhibitors of OPA1, 50 μM Myls22 (MedChemExpress) and Glucose-6-phosphate dehydrogenase, 50 μM G6PDi-1 (Sigma Aldrich) were added directly before the start of the swarming experiments. The CXCR2 antagonist, 5 μM SB225002 (Tocris), the inhibitor for the 5-lipoxygenase activating protein, 10 μM MK-886 (abcam), the inhibitor of mitochondrial fission, 10 μM Mdivi-1 (Sigma-Aldrich) and the mitochondrial fusion promotor, 20 μM M1 (Sigma Aldrich), were added 30 min before the start of swarming experiments. The Arp2/3 complex inhibitor CK-666, 100 μM CK-666 (Merck Millipore) and its inactive control, 100 μM CK-689 (Merck Millipore), were added 20 min before the start of swarming experiments.

#### Imaging of neutrophil swarms *in vitro*

Live cell imaging of murine neutrophils was performed using a LSM780 fluorescence confocal microscope equipped with a Plan Apochromat 63×/1.4 oil objective and ZEN black software (Carl Zeiss Microimaging). Fluorochromes were exited with solid-state lasers (UV405; Argon488, DPSS561, HeNe633). A stage-top incubator (Tokai-Hit) was used to create a humidified atmosphere with 5% CO_2_ at 37°C. Z-stacks were acquired in a range of 6–9 μm with a step size between 0.35 and 0.78 μm.

For inverted epifluorescence imaging of human neutrophils, 50 μL of the prepared neutrophil suspension was added to a well of the bioparticle array device, and a circular coverslip was placed atop the well, which created a seal and prevented the sample from drying out. This device was inserted into a fully automatic Nikon Ti2 microscope (Nikon, Tokyo, Japan) with a stage incubator (Okolab, Pozzuoli, Italy) set at 5% CO_2_ and 37°C. Time-lapse images of neutrophil crowding could then be recorded with desired brightfield and fluorescent channels. Fluorescence could be imaged through filters at: 405 nm (Hoechst 33342, DAPI), 488 nm (AlexaFluor 488), 555 nm (AlexaFluor 555), and 647 nm (AlexaFluor 647, FastAct). For live cell confocal imaging of human neutrophils, the device was prepared in the same manner as outlined above, but then was added to a Nikon A1R Live Cell Imaging Confocal Microscope (Nikon, Tokyo, Japan) that was equipped with climate control set at 37°C and 5% CO_2_ and filters utilized for 405 nm (Hoechst 33342), 488 nm (AlexaFluor 488), and 647 nm (AlexaFluor 647 and FastAct). For the fixed samples of human neutrophil clusters, the device was prepared as outlined in the immunofluorescence section and was added to an Olympus FV 3000 Confocal System (Olympus, Tokyo, Japan) with filters utilized for 405 nm (DAPI), 488 nm (AlexaFluor 488), 555 nm (AlexaFluor 555), and 647 nm (AlexaFluor 647).

#### Laser damage and imaging of neutrophil swarms *in vivo*

*In vivo* swarming upon laser-induced tissue damage was performed as described previously.^6^ The 5 μL dye-labeled neutrophil suspension was injected intradermally into the ventral side of ears of *Tyr*^*c−2J/c−2J*^ (C57BL/6-Albino) recipient mice. Then, 2–3 h after injection, mice were anesthetized with isoflurane. Mice were positioned in a temperature-controlled chamber in a way that placed the ventral ear pinna on a coverslip above the objective, where it was fixed. Tissue damage was done using a Chameleon XR Ti:Sapphire laser (Coherent) at 850 nm and 80 mW. A circular region with 15–25 μm diameter was scanned with pixel dwell time for 0.8 μs for 35–50 iterations. Imaging of neutrophils started directly after the laser damage using a 25×/0.8 NA Plan-Apochromat objective with glycerol as immersion medium on an inverted LSM 510 NLO multiphoton microscope (Carl Zeiss Microimaging) with AIM software. In Figure 1C neutrophils isolated from bone marrow of Tg(Lifeact-GFP) mice were injected into the skin of Tg(dsRed)*Tyr*^*c−2J/c−2J*^ host mice, before a local laser injury induced neutrophil clustering.^6^

#### Image analysis

Images and movies were processed using Imaris software (version 9.5, Bitplane). Swarm size was measured by manually counting cells, or nuclei of cells in experiments with Hoechst-staining, that participate in the swarm. The number of dead cells (Sytox positive) in and outside of swarms was manually counted per field of view (150 μm × 150 μm). Particle uptake was calculated based on images taken little above the surface using the Imaris spot function to count the number of particles over time. Frames, which shifted out of focus, were excluded from the analysis. Production of ROS over time (Figure 7) was calculated based on the MFI using Fiji (ImageJ2 version 2.9.0).^56^ Thereby, regions of interest were defined as “inside swarm” (a circle including all neutrophils above the particle pattern) and “outside swarm” (remaining image). The frequency of ROS signal within and outside of swarms was calculated using the spot function in Imaris (Figure 6). The distance of ROS signal located outside of the HKSA border to the HKSA border was measured using Fiji. The height of ROS signal was calculated using the surface function in imaris. Thereby, the bottom plane was defined using the HKSA signal and the imaged volume had a height of 14 μm. Mitochondrial size was quantified by rendering surfaces based on the MitoTracker channel with Imaris. Areas smaller 0.1 μm^2^ were regarded as noise signal and excluded from analysis. Membrane blebs were quantified by manually counting the number of cells with blebs within a swarm based 3D images.
