# Supplementary material for: Arp2/3 complex and the pentose phosphate pathway regulate late phases of neutrophil swarming
Source: iScience. 2023 Dec 16;27(1):108656. doi: 10.1016/j.isci.2023.108656 (PMC10777075; doi:10.1016/j.isci.2023.108656)
Supplement: Document S1. Figures S1–S4 [file mmc1.pdf]

iScience, Volume *27*

Supplemental information

Arp2/3 complex and the pentose phosphate pathway regulate late phases of neutrophil swarming

Katharina M. Glaser, Jacob Doon-Ralls, Nicole Walters, Xilal Y. Rima, Angelika S. Rambold, Eduardo Réategui, and Tim Lämmermann

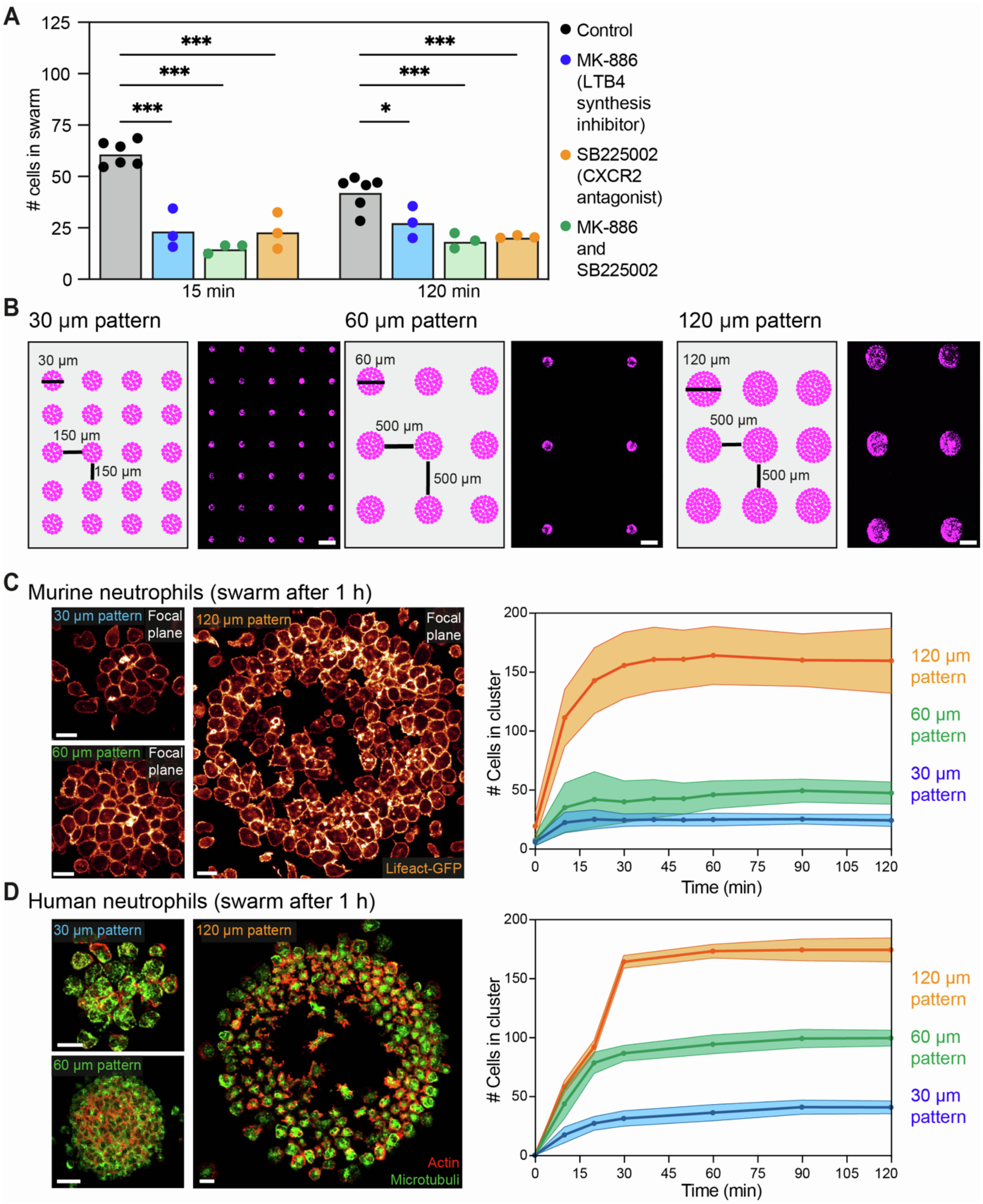

Figure S1. Characterizing the role of CXCL2, LTB4 and pattern size for swarming

(A) Quantification of swarm size after 15 min and 120 min. Mouse neutrophils were treated with SB225002, an CXCR2 antagonist, MK-886, an inhibitor of leukotriene biosynthesis, or both. n = 3 independent experiments with N = 5 swarms per condition. Two-way ANOVA, *$P < 0.05$, ***$P < 0.001$.

(B) Fluorescent HKSA particles (pink) were patterned as circles of 30 µm (left), 60 µm (middle), or 120 µm (right) diameter on glass coverslips. Left: Scheme. Right: Image of fluorescent particles.

(C) Swarm cluster growth of Lifeact-GFP expressing mouse neutrophils (glow) after 1 h on patterns of 30 µm, 60 µm, and 120 µm diameter, respectively (left). Scale bar: 10 µm. Quantification of cell numbers in growing neutrophil clusters (right). Data from n = 3 independent experiments with N = 4–6 swarms per condition. Data points display the mean (solid line) with SD (shade).

(D) Swarm cluster growth of human neutrophils stained against β-tubulin (green) and F-actin (red) after 1 h on patterns of 30 µm, 60 µm, and 120 µm diameter, respectively (left). Scale bar: 10 µm. Quantification of cell numbers in growing neutrophil clusters (right). Data from n = 3 independent experiments with N = 4–6 swarms per condition. Data points display the mean (solid line) with 95% confidence interval (shade).

Related to Fig. 1.

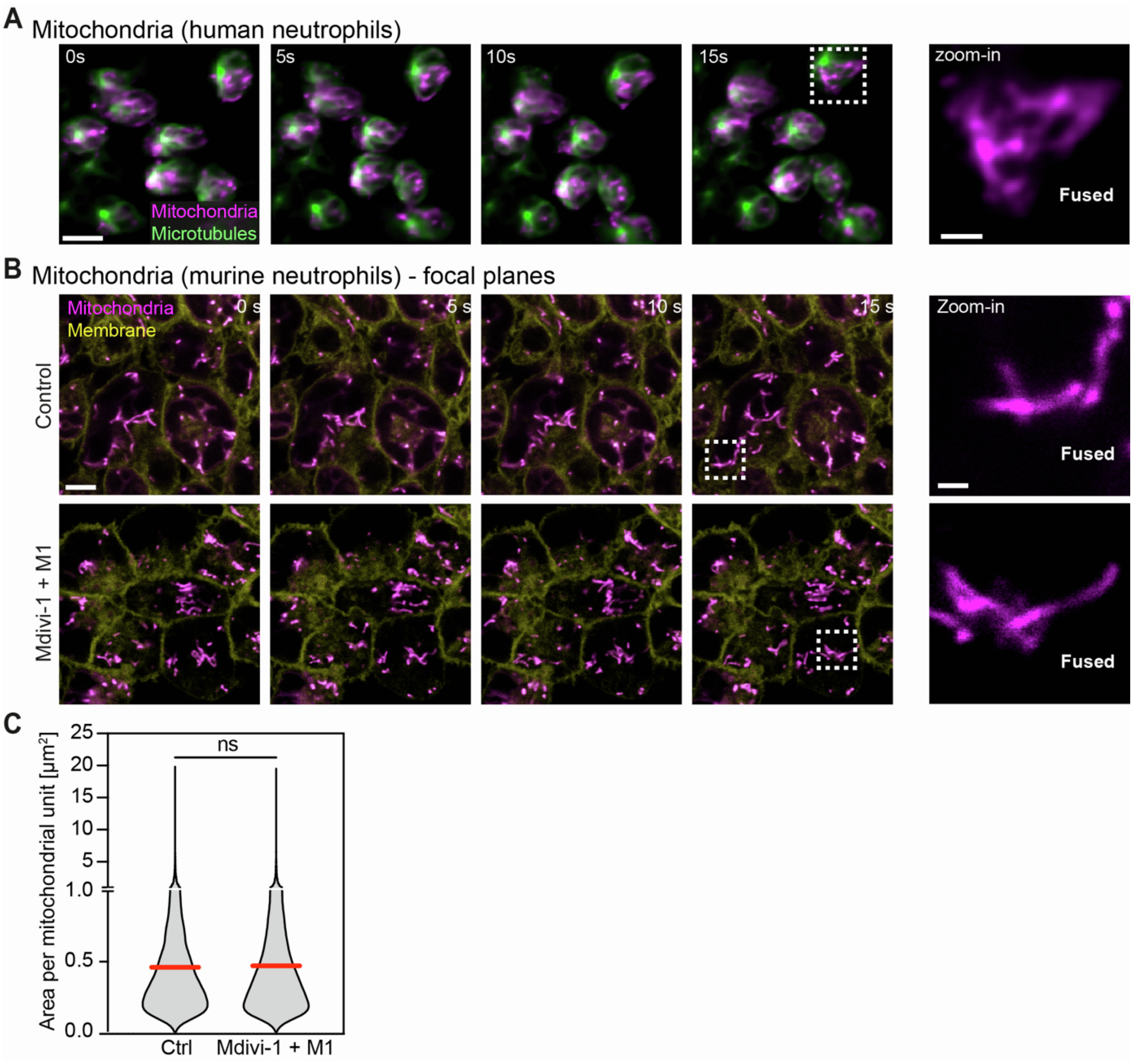

Figure S2. Fused mitochondria in swarming neutrophils

(A) Visualization of mitochondria in living swarming human neutrophils. Mitochondria are stained with MitoTracker™ Green (magenta), and microtubules are co-stained for orientation (green). Scale bar: 10 µm (time course) or 2 µm (zoom-in).

(B) Live cell imaging of mitochondrial dynamics in individual cells of crowding mouse neutrophils over time. Mitochondria are stained with MitoTracker™ Green (magenta), and neutrophils express membrane-tagged TdTomato (yellow). Cells were untreated (upper row) or treated with a combination of the mitochondrial fission inhibitor Mdivi-1 and the mitochondrial fusion promoter M1 (lower row). Zoom-in images show elongated mitochondria. Scale bar: 5 µm (time course) or 1 µm (zoom-in).

(C) Quantification of mitochondrial size in control and Mdivi-1 + M1 treated mouse neutrophils. Data from n = 3 independent experiments with in total N = 10500 (control) or 10142 (Mdivi-1 + M1) mitochondria. Red bars indicate the median. Mann-Whitney test, ns = not significant.

Related to Fig. 2.

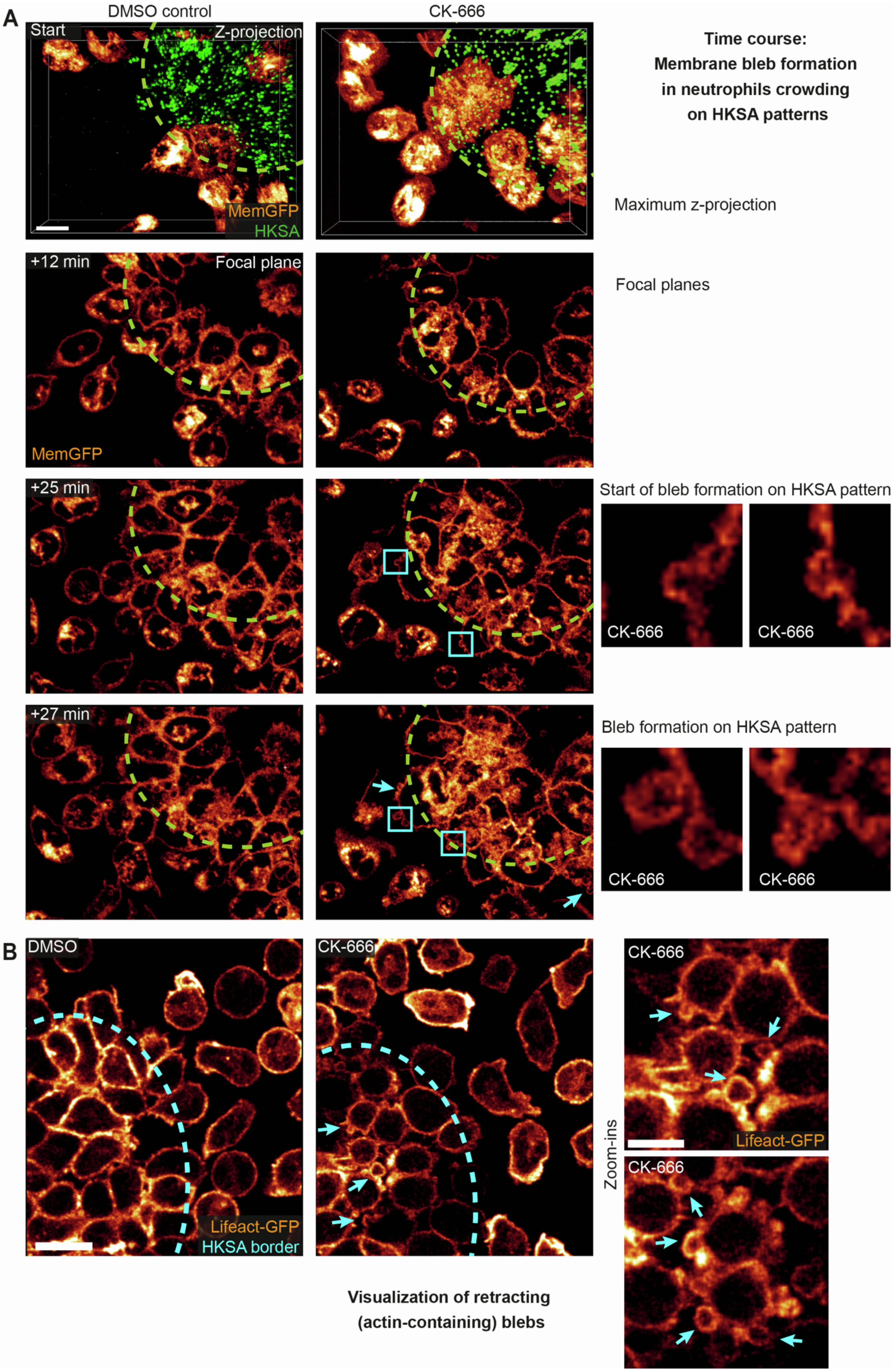

Figure S3: Arp2/3 complex inhibition causes blebs of the actin cytoskeleton

(A) Time course of neutrophil cluster formation with focus on the development of membrane blebs. Membrane-tagged GFP (glow) expressing mouse neutrophils were visualized over time, comparison of control with CK-666 treated neutrophils. The HKSA (green) border is displayed for orientation. Zoom-ins depict membrane blebs. Scale bar: 5 µm.
(B) Visualization of cortical actin and actin-containing projections in crowding Lifeact-GFP (glow) expressing neutrophils. CK-666 treatment leads to the appearance of actin-containing retracting blebs. Circles indicate HKSA borders. Arrows indicate blebs. Scale bar: 10 µm (overview) or 5 µm (zoom-in). Related to Fig. 3.

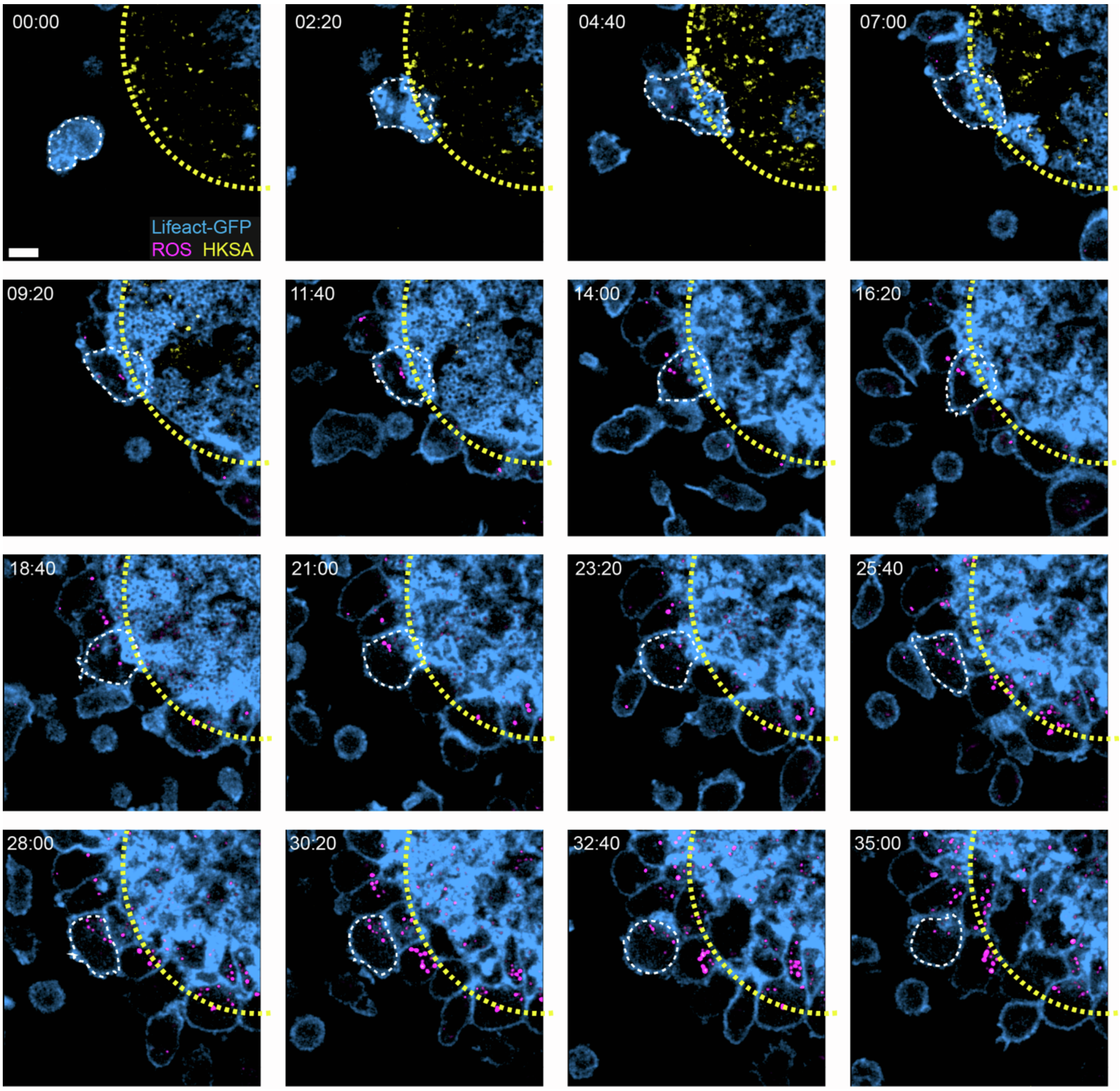

Figure S4: Example of ROS-producing cell exclusion from a developing neutrophil cluster

Time course displaying the positioning and onset of ROS production (magenta) of an individual Lifeact-GFP (blue) expressing mouse neutrophil over time. In this example, an early-arriving neutrophil makes contact with HKSA particles (02:20), starts to form phagocytic cup-like interactions with the HKSA patterns (04:40), starts to produce ROS (09:20) and becomes part of a growing neutrophil cluster. At late phases of neutrophil crowding, this ROS-producing cell is pushed out from the developing cluster (starting 23:20). The HKSA (yellow) border is indicated for orientation. Scale bar: 5 µm. Related to Fig. 6.
